# The prevalence of *Campylobacter* spp. in vegetables, fruits, and fresh produce: a systematic review and meta-analysis

**DOI:** 10.1186/s13099-018-0269-2

**Published:** 2018-09-27

**Authors:** Hooriyeh Mohammadpour, Enayat Berizi, Saeid Hosseinzadeh, Majid Majlesi, Morteza Zare

**Affiliations:** 1 Department of Food Hygiene, Faculty of Veterinary Medicine, Shahid Chamran University of Ahvaz, Ahvaz, Iran; 2 Nutrition Research Center, Department of Food Hygiene and Quality Control, School of Nutrition and Food Sciences, Shiraz University of Medical Sciences, Shiraz, Iran; 3 Department of Food Hygiene and Public Health, School of Veterinary Medicine, Shiraz University, Shiraz, 71345-1731 Iran; 4 Department of Nutrition Sciences, School of Health and Nutrition Sciences, Yasuj University of Medical Sciences, Yasuj, Iran

**Keywords:** Campylobacter, Raw vegetable, Foodborne infections

## Abstract

There are a number of reports indicating correlation between outbreaks of campylobacteriosis and the consumption of raw vegetables. This study is a meta-analysis on the prevalence of *Campylobacter* in fresh vegetables and fruits without any location limitation, which was performed through a documented review of the available resources. Relevant literature was reviewed by trained reviewers, who examined the results for the inclusion of articles in the meta-analysis. The prevalence of *Campylobacter* in raw vegetables, the sample source, the *Campylobacter* species, and the method of detection were extracted. The prevalence of *Campylobacter* in vegetables, fruits, and fresh produce were estimated to be 0.53%. Analysis of the various sample groups initially showed that the bean and sprouts group was the vegetable with the highest prevalence of *Campylobacter* (11.08%). The rate of contamination was higher when both the molecular and conventional methods were employed. The highest prevalence of *Campylobacter* was found in Asia (33.4%). Despite the low prevalence, consumption of raw vegetables is inherently risky because no treatment is used to inactivate the pathogens. Therefore, proper sanitation methods are recommended to treat the raw products.

## Background

In recent years, it is emphasized that consuming the organic food is associated with a healthier lifestyle. Thus, new food consumption trends indicate that people are interested in freshly produced organic foods. Among them, the consumption of fresh cut or minimally-processed fruit and vegetables have undergone a sharp increase. Such trends have been reflected in an increase in the popularity of salad bars in many countries [1–3]. In terms of retail, vegetables can be sold intact or minimally processed to provide a ready-to-eat product and can be contaminated at any point in the chain, starting from the farm to the plate. As they are not subjected to any treatment to eliminate pathogens, a diverse range of human enteric pathogens can contaminate them. There are a number of reports showed the correlation between foodborne illness outbreaks and the consumption of raw vegetables, annually [4, 5]. Several bacterial pathogens have been implicated in foodborne illnesses associated with the consumption of raw vegetables, such as *Salmonella* spp., thermo-tolerant *Campylobacter*, *Listeria monocytogenes*, and certain enteric viruses [6]. These may contaminate vegetables during any stage of production. The yearly average frequency of foodborne outbreaks linked with fresh produce contamination between 2002 and 2012 was reported by Wadamori et al. [7] with the prevalence of 57% (USA), 8% (Japan), and 6% (New Zealand). Infection by *Campylobacter* spp., specifically *Campylobacter jejuni* and *Campylobacter coli,* are the major cause of the mild bacterial diarrhea disease in the world [8]. *Campylobacter* spp. is estimated as the third most common bacterial cause of foodborne illness, but relatively few outbreaks have been detected [5]. Studies in high-income countries have estimated the annual incidence between 4.4 and 9.3 per 1000 population. While, the disease is usually self-limiting within 3–7 days, an acute infection can have serious long-term consequences, including severe neurological dysfunctions, such as Guillain–Barré syndrome (GBS) and Miller Fisher syndrome (MFS), and functional bowel diseases, such as irritable bowel syndrome (IBS) [9]. In 2013, the overall national incidence of campylobacteriosis infections per 100,000 population was estimated to be 6.621, which led to 1010 hospitalizations and 12 death [10]. In 2011, the Euro surveillance editorial team reported that out of a total of 5048 outbreaks of foodborne diseases, *Campylobacter* was responsible for 220,209 cases which occurred in the European Union (EU) [11]. It has been estimated that 75% [12] and 82% [13] of *Campylobacter* disease in Australia was associated with food. Most fruits and many vegetables are typically consumed raw and may also be as an important vehicle for *Campylobacter* spp. It is essential to assess *Campylobacter* as a relevant microbial risk for raw vegetables, fruits and minimally processed packaged salads, because can be pail of the indigenous microflora of fresh produce. A number of reports refer to fresh produce harboring potential foodborne pathogens. Lettuce and spinach are described in the international literature as the main vegetable sources of human infection by *Campylobacter* spp. [1, 16, 25, 26]. An increased interest in the campylobacteriosis risk assessment of raw vegetables is driven by several outbreaks of infections caused by consumption of fresh produce, such as leafy vegetables and salads [14], lettuce [15], and sprout and cabbage [16]. Studies have revealed that travelling to Asia, Africa, Latin America, the Caribbean, and Southern Europe significantly increased the risk of acquiring campylobacteriosis as compared to travelling within Western Europe [17–19]. Between 2004 and 2012, total of seven and three outbreaks of campylobacteriosis associated with the consumption of fresh vegetables have occurred in the United States and Europe, respectively [20]. Studies such as Evans et al. [21]; Mellou et al. [22] and Danis et al. [3] reported that fresh vegetables and fruits could be considered as risk factors for *Campylobacter* infection.

Role of fresh vegetable as a risk factor in campylobacteriosis, was previously addressed. Previous studies reported different prevalence of infection in assorted fresh vegetables. Present systematic review and meta-analysis study was aimed to focus on the more precise prevalence of infection. Therefore our study will be useful to find out the role of each vegetable to cause the infection.

## Methods

### Search strategy

A comprehensive scientific search on the presence of *Campylobacter* spp. in freshly produced food was carried out in three valid electronic global databases: PubMed, Scopus, and Science Direct using the same keywords. The search was performed through systematic research from the year 1990 till 2017. Keywords used to filter through the databases were: *Campylobacter*, vegetable, lettuce, spinach, leafy vegetable, sprout, fruits, salad, rocket, onion, carrot, cilantro, tomato, cucumber, broccoli, cabbage, cantaloupe, parsley, arugula, pepper, blueberry, strawberry, apple, peach, and melon. Articles containing any of these keywords in their abstracts or titles were included. A total of 135 articles were finally selected.

### Study selection

After screening these relevant abstracts, 80 articles were selected. Articles that did not use the English language in the main text, review articles, and book chapters, as well as publications, related to the surveillance of case control study, risk factors, outbreaks of campylobacteriosis, genotyping, food handlers with their hygienic practices, and artificially contaminated samples were excluded from the study. Thereafter, full text screening of all the eligible primary studies was carried out from the databases. In case that full text of the articles were not available, they were finally excluded. To improve the reliability, our included articles was screened by two independent researchers.

### Data extraction

Population of the study included vegetables, fruits, and freshly produced food investigated in each relevant primary study. Food that has been considered as fresh produces in this study are vegetables [fresh cut, organic, leafy, root crops, and ready-to-eat (RTE)], beans and sprouts, salad (mixed, gravy), and fruits (fresh cut, mixed, or fruit crops). Various samples were collected from restaurants, retail shops, farm, supermarkets, and ready-to-eat street-vended foods. Studies that apply any treatment, such as heat, pressure, irradiation, and bactericidal on fresh produce, and those found to report effects of cross-contamination were disregarded from the assay. Different kinds of salads and vegetables were categorized into a few subgroups.

### Statistical analysis

All the data was analyzed using the Stata^®^ 13.0 software (StataCorp LP., College Station, Texas, USA). Confidence interval of the prevalence rate of *Campylobacter* spp. in every study was calculated on the basis of binomial proportion formula. Statistical heterogeneity was assessed with the help of the I_2_ and Chi square test. For heterogeneity recognition, p < 0.05 and I square > 50%. Random-effects model was used to calculate the prevalence estimate after the heterogeneity test.

## Results and discussion

### Systematic review

#### Search results and selection of studies

**Fig. 1:**
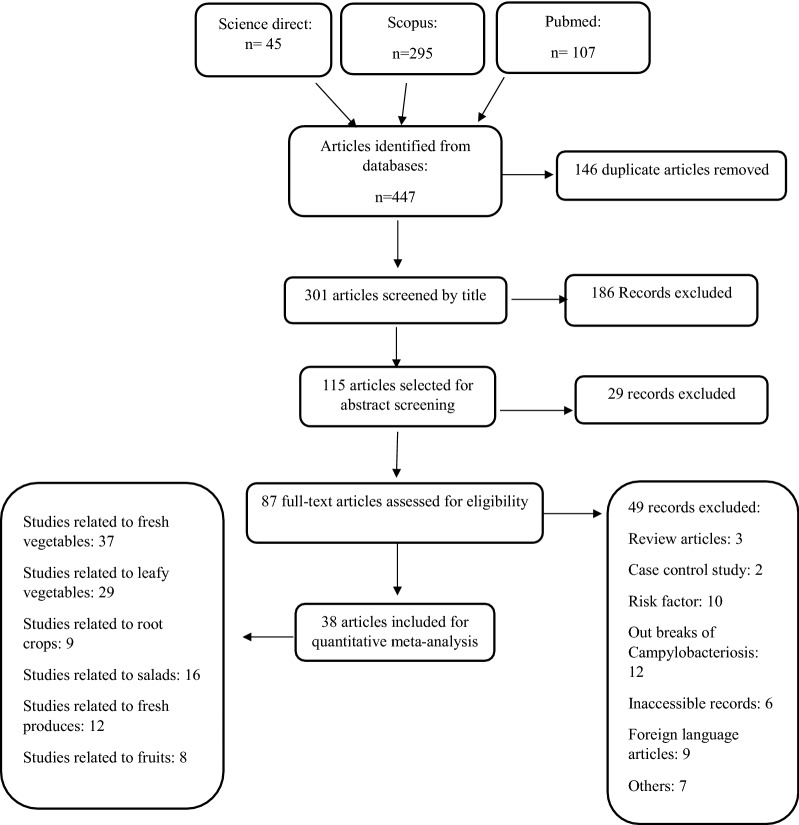
Flowchart stages of the entry studies into a systematic review and meta-analysis

Following research using electronic global databases, a list of titles and abstracts from all the articles provided by the researcher was evaluated independently based on the selected keywords and elimination of similar articles in order to determine and select related topics. From a total of 447 records, at least 301 studies selected as related articles. These articles were assessed by their titles; 115 articles were included. After screening of relevant abstracts, full text of 87 articles were obtained and assessed for eligibility. Out of these, 49 studies were excluded based on inclusion and exclusion criteria mentioned in the methodology. Considering all the requirements, at least 38 studies were finally included in the quantitative meta-analysis. Some studies related to basic scientific, quality, quantity, and methodologies were selected for additional assessment (Fig. 1). All the selected articles were classified based on total samples, prevalence, commodity, isolation method, and region, and were collected for the preparation of a check list by the researcher. Sample collections were grouped into seven categories: vegetables, RTE vegetables, leafy vegetables, root crops, salad, beans and sprouts, and fruit and evaluated using two dimensions of scientific principles and methodology accuracy.

#### Characteristics of studies and data extraction

The summary plan of this study has been presented in Table 1. Although in most studies the prevalence of *Campylobacter* was low, the highest prevalence of *Campylobacter* spp. was reported by Khalid et al. [16]. Out of the seven food categories, freshly produced food showed the highest prevalence, while the lowest rate of contamination was associated with the consumption of salads. Among *Campylobacter* species, *C. jejuni* has reflected the highest prevalence in targeted population, while only one study confirmed the isolation in lettuce. The major detection methods were included the selective culture, molecular, and a combination of culture/molecular techniques. The presence of pathogen was confirmed by of the selective culture method (n = 29). Thirty studies were performed to isolate different species of *Campylobacter* regardless of any limitation. This analysis revealed seven researches in Asia, three in Africa, nineteen in Europe, one in Oceania, two in South America, and six in North America.

**Table 1:** Information of included studies in the meta-analysis of prevalence of *Campylobacter* spp. in vegetables, fruits and fresh produces

References	N^a^	n^b^	P (%)	95% Cl	*Cam.Sp*	Sample	Method	Country	V (g)^c^
[1]	5	0	0	0–49.06	spp.	Arugula	Culture	Spain	25
	18	0	0	0–20.95	spp.	Carrot	Culture	Spain	25
	21	0	0	0–18.63	spp.	Corn salad	Culture	Spain	25
	21	0	0	0–18.63	spp.	Endive	Culture	Spain	25
	29	0	0	0–14.1	spp.	Lettuce	Culture	Spain	25
	10	0	0	0–32.17	spp.	Spinach	Culture	Spain	25
	15	0	0	0–21.28	spp.	Sprouts	Culture	Spain	25
	132	0	0	0–3.37	spp.	Mixed salads	Culture	Spain	25
	21	0	0	0–18.63	spp.	Fresh–cut fruit	Culture	Spain	25
	28	0	0	0–14.63	spp.	Whole vegetables	Culture	Spain	25
[23]	40	0	0	0–9.75	spp.	Fresh vegetable	Culture	Austria	25
	36	0	0	0–11.75	spp.	Mixed salad	Culture	Austria	25
[24]	128	0	0	0–3.58	spp.	Lettuce	Culture	Canada	25
	59	0	0	0–7.37	spp.	Spinach	Culture	Canada	25
	129	0	0	0–3.56	spp.	Green onions	Culture	Canada	25
	206	0	0	0–2.26	spp.	Carrots	Culture	Canada	25
	120	0	0	0–3.8	spp.	Tomatoes	Culture	Canada	25
	31	0	0	0–13.38	spp.	Strawberry	Culture	Canada	25
[25]	40	2	5	0–11.75	*jejuni*	Lettuce	Molecular	Brazil	25
	40	1	2.5	0–7.33	*coli*	Lettuce	Molecular	Brazil	25
	40	0	0	0–10.62	spp.	Spinach	Molecular	Brazil	25
[26]	80	0	0	0–4.6	spp.	Strawberry	Culture/molecular	Belgium	25
	241	8	3.3	1.7 –6.4	spp.	Leafy greens	Culture/molecular	Belgium	25
[8]	40	4	10	0.7–19.3	spp.	Yard long bean	Culture/molecular	Malaysia	10
	39	18	46.1	31–61	spp.	Winged bean	Culture/molecular	Malaysia	10
	41	23	56.09	41–71	spp.	Mung bean sprout	Culture/molecular	Malaysia	10
	36	20	55.5	40–70	spp.	Vietnamese coriander	Culture/molecular	Malaysia	10
	39	21	53.8	38–70	spp.	Japanese parsley	Culture/molecular	Malaysia	10
	37	22	59.4	43–74	spp.	Indian pennywort	Culture/molecular	Malaysia	10
	38	13	34.2	19–49	spp.	Wild cosmos	Culture/molecular	Malaysia	10
[27]	49	4	8.16	0–15.7	spp.	Vegetable from farm	Culture/molecular	Malaysia	10
[28]	27	0	0	0–14.3	*jejuni*	Vegetable	Culture	Vietnam	250
[29]	5170	0	0	0–0.09	spp.	Leafy vegetables	Culture	Canada	25
	3696	0	0	0–0.13	spp.	Leafy herbs	Culture	Canada	25
[30]	400	2	0.5	0.0–1.2	*jejuni*	Grated vegetables	Culture/molecular	France	20
[31]	50	1	2	0.0–5.88	spp.	Parsley	Culture/molecular	Mexico	25
[32]	88	8	9	3.02–14.97	spp.	Lettuce	Culture	Belgium	25
[15]	48	4	8.3	0.5–16.1	spp.	Greenhouse lettuce	Culture	Belgium	25
	40	4	10	0.7–19.3	spp.	Open field farm lettuce	Culture	Belgium	25
[33]	22	9	40.9	19.52–60.47	*jejuni*	Vegetable/fruit salads	Culture	Pakistan	10
[34]	80	0	0	0–5.5	spp.	Strawberry	Culture/molecular	Norway	10
[16]	61	22	36.06	24–48	*jejuni*	Winged bean	Culture/molecular	Malaysia	10
	60	40	66.6	54–78	*jejuni*	Long yard bean	Culture/molecular	Malaysia	10
	20	11	55	34–76	*jejuni*	Indian pennywort	Culture/molecular	Malaysia	10
	47	20	42.5	28.4–56.6	*jejuni*	Japanese parsley	Culture/molecular	Malaysia	10
	10	7	70	42–98	*jejuni*	Vietnamese coriander	Culture/molecular	Malaysia	10
	23	12	52.2	31.6–72.4	*jejuni*	Cucumber	Culture/molecular	Malaysia	10
	30	21	70	54–86	*jejuni*	Cabbage	Culture/molecular	Malaysia	10
	10	8	80	56–104	*jejuni*	Mung bean sprout	Culture/molecular	Malaysia	10
	70	50	71.4	70–81.9	*jejuni*	Wild cosmos	Culture/molecular	Malaysia	10
[35]	9	1	11.11	0–31.44	*jejuni*	Spinach	Culture	India	25
	9	1	11.11	0–31.44	*jejuni*	Fenugreek	Culture	India	25
	9	0	0	0–34.86	spp.	Cauliflower	Culture	India	25
	9	0	0	0–34.86	spp.	Cabbage	Culture	India	25
	10	0	0	0–32.48	spp.	Coriander	Culture	India	25
	4	0	0	0–55	spp.	Raddish	Culture	India	25
	6	0	0	0–44.79	spp.	Carrot	Culture	India	25
[36]	151	0	0	0–2.9	spp.	Lettuce	Culture	UK	25
[37]	1372	12	0.9	0.4–1.4	spp.	Fresh leafy vegetable	Culture/molecular	Italy	25
	1160	6	0.5	0.1–0.9	spp.	Ready to Eat vegetable	Culture/molecular	Italy	25
[38]	86	0	0	0–5.23	spp.	Organic vegetable	Culture	North Ireland	25
[39]	42	0	0	0–10.16	spp.	RTE vegetables	Culture	Canada	100
[40]	1260	0	0	0–0.36	spp.	Fruit and vegetables	Culture	UK	25
	224	0	0	0–2.07	spp.	Mixed salads	Culture	UK	25
	226	0	0	0–2.05	spp.	Coleslaw (Salad)	Culture	UK	25
[41]	12	0	0	0–28.7	spp.	Salad	Culture	South Africa	25
[42]	22	0	0	0–17.78	*jejuni*	Salad/gravy prepared	Culture	South Africa	20
	22	0	0	0–17.78	*jejuni*	Salad/gravy during holding	Culture	South Africa	20
	22	0	0	0–17.78	*jejuni*	Salad/gravy raw materials	Culture	South Africa	20
[43]	65	0	0	0–6.85	spp.	RTU vegetables	Culture	Canada	25
	296	0	0	0–1.47	spp.	RTU vegetable	Culture	Canada	25
[44]	183	2	1.09	0–2.4	spp.	Spinach	Culture	Canada	50
	348	2	0.57	0–1.24	spp.	Lettuce	Culture	Canada	50
	174	2	1.15	0.0–2.65	spp.	Radish	Culture	Canada	200
	160	1	0.62	0–1.8	spp.	Green onion	Culture	Canada	50
	177	1	0.56	0–1.54	spp.	Parsley	Culture	Canada	50
	153	1	0.65	0.0–1.82	spp.	Potatoes	Culture	Canada	200
	150	0	0	0.0–3.09	spp.	Celery	Culture	Canada	50
	130	0	0	0.0–3.55	spp.	Cabbage	Culture	Canada	200
	149	0	0	0–3.09	spp.	Carrot	Culture	Canada	200
	123	0	0	0.0–3.61	spp.	Cucumber	Culture	Canada	200
	482	14	2.9	1.5–4.5	spp.	Fresh vegetables	Culture	Canada	50/200
[45]	90	20	22.2	13.5–30.5	spp.	MAP mixed salad	Culture	UK	10
[46]	2870	0	0	0–0.165	spp.	RTE salads	Culture	UK	25
[47]	3852	0	0	0–0.122	spp.	RTE salad vegetables	Culture	UK	25
[48]	3200	0	0	0–0.148	spp.	RTE organic vegetables	Culture	UK	25
[49]	94	0	0	0–4.93	spp.	Chicken salad	Culture/molecular	UK	25
	35	0	0	0–12	spp.	Ham salad	Culture/molecular	UK	25
	12	0	0	0–28.7	spp.	Salmon salad	Culture/molecular	UK	25
[50]	28	0	0	0–14.6	*jejuni*	Vegetable	Culture	Malawi	10
[51]	40	0	0	0–10.6	spp.	Vegetable	Culture	United States	25
[52]	11	1	9.1	0–25.9	*jejuni*	Cucumber	Culture	Malaysia	25
	9	0	0	0–34.8	*jejuni*	Lettuce	Culture	Malaysia	25
[53]	55	0	0	0–7.85	*jejuni*	Asparagus	Culture	New Zealand	50
	55	0	0	0–7.85	*jejuni*	Mung bean sprouts	Culture	New Zealand	50
	55	0	0	0–7.85	*jejuni*	Watercress	Culture	New Zealand	50
	55	0	0	0–7.85	*jejuni*	Spinach	Culture	New Zealand	50
	55	0	0	0–7.85	*jejuni*	Silver beet	Culture	New Zealand	50
[14]	1157	2	0.17	0.02–0.62	spp.	Fruit crops	Culture	Netherland	25
	196	0	0	0–1.86	spp.	Root crops	Culture	Netherland	25
	127	0	0	0–2.86	spp.	Cabbage	Culture	Netherland	25
	8	0	0	0–36.94	spp.	Mushrooms	Culture	Netherland	25
	42	0	0	0–8.41	spp.	Onions, garlic	Culture	Netherland	25
	50	1	2	0.05–10.65	spp.	Stem and sprout crops	Culture	Netherland	25
	2549	5	0.2	0.06–0.46	spp.	Mixed salads/vegetables	Culture	Netherland	25
	159	1	0.6	0.02–3.45	spp.	Vegetable-fruit mix	Culture	Netherland	25
	11	0	0	0–28.49	spp.	Fruit	Culture	Netherland	25
	779	2	0.3	0.03–0.92	spp.	Mixed fruit	Culture	Netherland	25
	562	2	0.36	0.04–1.28	spp.	Leafy vegetables	Culture	Netherland	25
[54]	217	2	0.9	0.0–2.2	*jejuni*	Mushrooms	Culture	Ireland	10
	62	0	0	0–7.11	spp.	Vegetables/salad	Culture	Ireland	10
[55]	1810	3	0.22	0.06–0.48	spp.	Raw vegetable	Culture	Netherland	25
	764	0	0	0–0.5	spp.	Vegetable	Culture	Netherland	25
	1151	0	0	0–0.4	spp.	Vegetable	Culture	Netherland	25

^a^ Number of samples, ^b^ Number of positive samples, ^c^ Sample volume

### Meta-analysis results

#### Overall prevalence

**Fig. 2:**
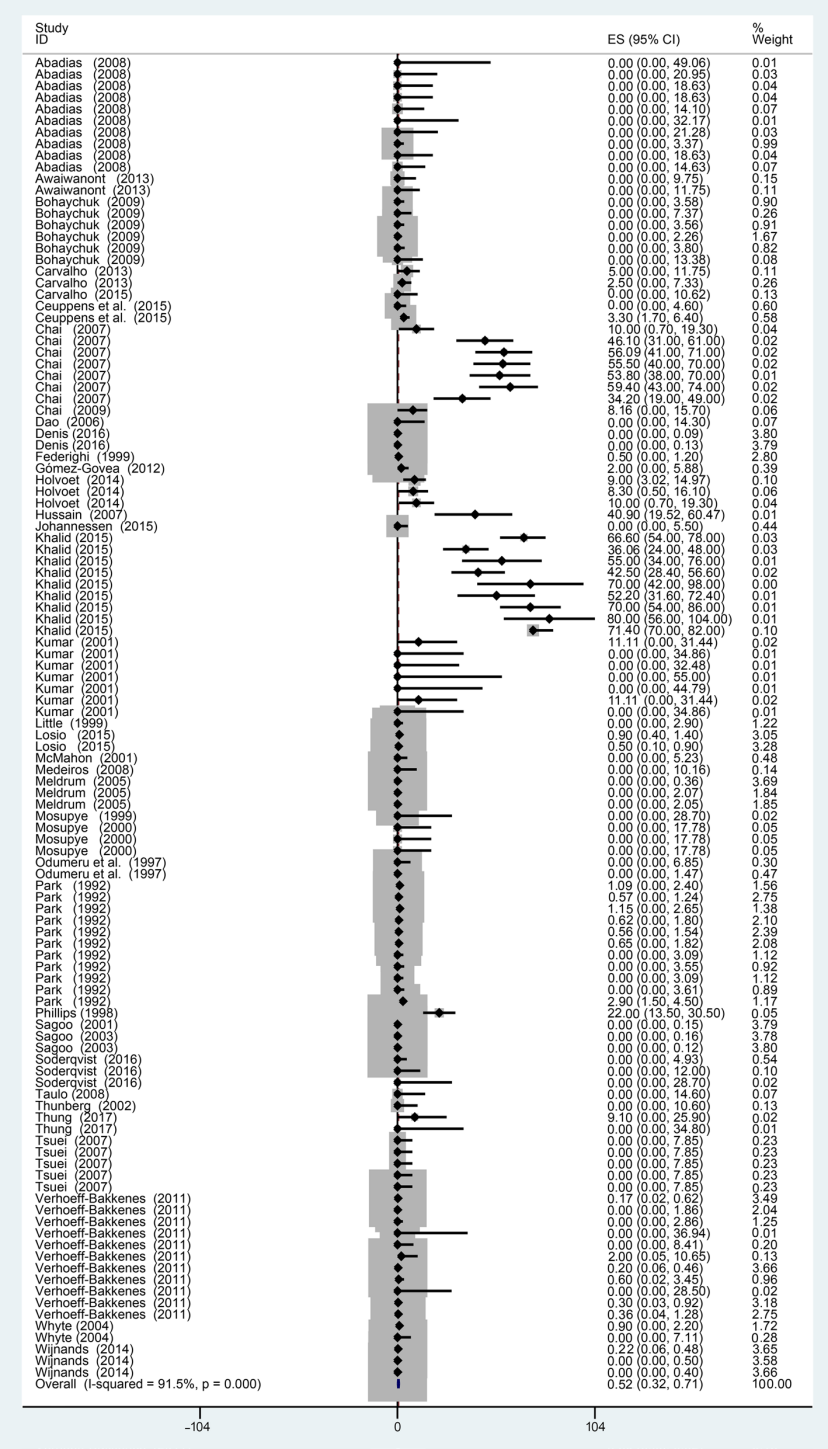
Forest plot for meta-analysis of *Campylobacter* spp. prevalence

The total prevalence of *Campylobacter* in vegetables, fruits, and fresh products was estimated at 0.53% (Fig. 2). The results showed a low occurrence of *Campylobacter* based on the reports of Losio and Verhoeff-Bakkenes, where the prevalence was less than one percent in vegetables and fruits [30, 37]. Lower rates of isolation were probably due to problems in the growth and recovery of microorganisms. Based on many scientific research reports, foods of animal origin, such as raw milk [56], turkey, chicken, beef, pork [57] and manure [58] were considered as the major sources of *Campylobacter* spp. Hence, it is likely that the occurrence of *Campylobacter* spp. in the targeted resource of this study was due to cross-contamination during growth, irrigation, harvest, transportation, and further processing and handling. Danis and Pintar both supported this hypothesis [3, 59].

#### Type of samples

All of the target samples included for this review have been listed in Table 2. Fresh produce, in particular fruit, does not receive any lethal treatment that kills all pathogens prior to consumption. Results related to the prevalence of pathogen in the different types of produce subgroups have been presented in Table 3. The results of the meta-analysis demonstrated that, among the different group of samples, the beans and sprouts (11.08%) revealed the highest prevalence, followed by the vegetable, detected in 1.73% of samples from supermarkets, retails, and farm lands. The minimum prevalence of *Campylobacter* was belong to the salad and fruit, which estimated at around 0.02% and 0.20%, respectively. As shown in Table 2, the highest prevalence of *Campylobacter* was found in the Indian pennywort and wild cosmos. Fields on which livestock or wild animals have grazed are more likely to be contaminated with enteric pathogens. Factors, such as bacterial presence in livestock, companion animals, wild animals, insects, and the natural environment, including soil and surface waters, lack of good agricultural practices (GAP), and cross-contamination with manure, could be related to the presence of pathogens in these vegetables [60]. Also, high prevalence was found in beans and sprouts. Lots of outbreak reports throughout the world have been linked to the consumption of raw and lightly cooked sprouts [61, 62]. Sprout production involves a unique seed germination process that can support the growth of pathogens because its germination is ideal for bacterial proliferation [63]. Additional factors, such as nutritive value, root nature of sprout, cross-contamination by manure, and irritation might have influenced the microbial contamination of these products. When manure is spread on agricultural fields, it possibly goes into the surface water. Hence, along with weak good manufacturing practice (GMP) and GAP, the presence of environmental bacteria may occur in food. Low prevalence in salad vegetables (0.02%) and fruits (0.20%) may be due to the accurate and sufficient attention paid towards hygiene of salad commodities and also sensitivity to acidic conditions (pH < 5.0) for fruits. Human or animal sources, as well as handling in the stores, may also be associated with increasing the microorganisms at the surface of fresh produce. The low temperature and lack of nutrients at the surface of fruits cause a reduction in *Enterobacteriaceae* during storage. It can also be due to the breaking of the cold chain during shelf-life or handling by the shoppers. Therefore, it is not surprising to find *Campylobacter* on the surface of fresh produce [64].

**Table 2:** Meta-analysis of prevalence of *Campylobacter* in all of foods

Source^a^	Total inputs^b^	Total sample size^c^	Overall prevalence (%)	95% confidence interval	I^2^ (%)	P for χ^2^
Pennywort	2	57	57.84	45.37–70.31	0.00	0.74
Wild cosmos	2	108	53.46	17.02–89.89	95.10	0.00
Coriander	3	56	41.00	0.00–83.65	93.60	0.00
Bean	4	200	39.47	13.81–65.13	94.70	0.00
Sprouts	5	171	23.68	6.68–40.68	95.60	0.00
Parsley	4	313	18.58	8.54–28.62	96.10	0.00
Cucumber	3	157	18.30	0.00–42.00	92.50	0.00
Fenugreek	1	9	11.11	0.00–26.83	–	–
Cabbage	4	296	10.42	2.38–18.45	95.90	0.00
Lettuce	10	921	1.53	0.12–2.94	54.00	0.02
Radish	2	178	1.14	0.00–2.47	0.00	0.93
Spinach	6	356	0.91	0.00–1.98	0.00	0.81
Mushroom	2	225	0.89	0.00–1.99	0.00	0.92
Potato	1	153	0.65	0.00–1.56	–	–
Fresh cut vegetables	2	421	0.50	0.00–1.10	–	–
Green Onion	2	289	0.49	0.00–1.29	0.00	0.54
Fruits	4	1968	0.21	0.00–0.45	0.00	0.97
RTE vegetables	5	4763	0.13	0.00–0.40	31.00	0.21
Vegetables	15	8535	0.12	0.00–0.28	38.40	0.06
Leafy vegetables	5	11,041	0.10	0.00–0.25	81.10	0.00
Salad	16	7692	0.02	0.00–0.26	63.50	0.00
Onion	1	42	0.00	0.00–4.20	–	–
Crops	1	196	0.00	0.00–0.93	–	–
Beet	1	55	0.00	0.00–3.92	–	–
Water cress	1	55	0.00	0.00–3.92	–	–
Asparagus	1	55	0.00	0.00–3.92	–	–
Celery	1	150	0.00	0.00–1.54	–	–
Cauliflower	1	9	0.00	0.00–17.43	–	–
Strawberry	3	191	0.00	0.00–1.70	0.00	1.00
Tomatoes	1	120	0.00	0.00–1.90	–	–
Endive	1	21	0.00	0.00–9.31	0.00	0.00
Arugula	2	60	0.00	0.00–24.53	0.00	0.00
Carrot	4	379	0.00	0.00–0.90	0.00	1.00

^a^ Different type of fresh vegetables and fruits

^b^ Number of distinctive prevalence values is reported

^c^ Number of vegetable and fruit samples used to determine each estimate

**Table 3:** Prevalence of *Campylobacter* in subgroups of freshly produced foods

Source^a^	Total inputs^b^	Total sample size^c^	Overall prevalence (%)	95% confidence interval	I^2^ (%)	P for χ^2^
*Vegetables*						
Organic vegetable, asparagus, parsley, coriander, tomatoes, green onion, cucumber, endive, mushroom, arugula, cosmos, fenugreek, cauliflower, Celery	39	10,094	1.73	1.04–2.41	95.10	0.00
*RTE vegetables*						
Fresh cut vegetables, RTU and RTE vegetables	3	1602	0.49	0.16–0.83	0.00	0.98
*Leafy vegetables*						
Spinach, lettuce, cabbage, pennywort, water cress	29	12,726	0.49	0.17– 0.82	87.00	0.00
*Root crops*						
Radish, potato, carrot, beet	9	961	0.34	0.00–0.82	0.00	0.93
*Salad*						
MAP mixed salad, RTE salads, chicken salad, ham salad, salmon salad	16	7692	0.02	0.00–0.26	63.50	0.00
*Bean and sprouts*						
Winged bean, long yard bean, sprouts, mung bean sprout	12	3932	11.08	7.82–14.33	96.20	0.00
*Fruit*						
Fruits, strawberry, fruit salads	8	2168	0.20	0.00–0.45	0	1.00

^a^Sample collections were grouped into seven categories: vegetables, RTE vegetables, leafy vegetables, root crops, salad, beans and sprouts, and fruit

^b^Number of distinctive prevalence values is reported

^c^Number of vegetable and fruit samples used to determine each estimate

#### Campylobacter species

Results of the statistical analysis also showed that the highest prevalence of *Campylobacter* was observed for *C. jejuni,* with a percentage of 18.20%, whereas other *Campylobacter* spp. had the minimum prevalence, with a percentage of 0.23% (Table 4). Actually, among different species*, C. jejuni* showed the highest prevalence [54, 65]. It is worth mentioning that the aim of majority of the papers assessed in this study was to consider no specific species of *Campylobacter.* The highest prevalence of *Campylobacter* was identified by molecular approaches. *C. jejuni* mainly resided in the intestinal tract of warm-blooded animals and birds, and, therefore, the excreta may act as a source of contamination. Isolation of *C. jejuni* from vegetables was possibly due to the fecal contamination of these commodities and water at any step of the production chain. However, contact with the utensils used to process raw chicken was also important as they were the main reservoirs of *C. jejuni* [66]. In developed countries, *C. jejuni* was the most frequent cause of acute diarrheal infections. An improvement in the survival of *C. jejuni* in soil and rhizosphere is possibly a substantial factor in the environmental cycle of bacteria [67].

**Table 4:** Prevalence values and sample sizes for *Campylobacter* species provided in Table 1

Species^a^	Total inputs^b^	Total sample size^c^	Overall prevalence (%)	95% confidence interval	I^2^ (%)	P for χ^2^
*Campylobacter* spp.	86	37,682	0.23	0.11–0.35	77.8	0.000
*Campylobacter jejuni*	27	1444	18.20	13.63–22.77	97.2	0.000
*Campylobacter coli*	1	40	2.50	0.0–6.16	_	_

^a^Different species of *Campylobacter*

^b^Number of distinctive prevalence values is reported

^c^Number of vegetable and fruit samples used to determine each estimate

#### Methods of detection

Various isolation methods have been applied according to the literature. The results of the meta-analysis have shown on more than one method for better identification of the bacterium, and thus the estimated prevalence in this method was 21.52% (Table 5). Higher prevalence rates were reported using most probable number PCR (MPN-PCR) by Khalid et al. [16] and Chai et al. [8]. Additionally, there have been articles documenting the positive efficacy of this method for the isolation of food-borne pathogens in various food types. Norinaga et al. [68] compared two methods, MPN-PCR and MPN- thiosulfate citrate bile sucrose agar (MPN- TCBS agar), for the detection and enumeration of *Vibrio parahaemolyticus* in sea foods. The results showed that MPN-PCR was more convenient and reliable compared to MPN-TCBS, which was also supported by Luan et al. [69].

**Table 5:** Prevalence values and sample sizes for detection method of *Campylobacter*

Method^a^	Total inputs^b^	Total sample size^c^	Overall prevalence (%)	95% confidence interval	I^2^ (%)	P for χ^2^
Culture	85	34,922	0.06	0.01–0.12	23.7	0.03
Molecular	3	120	2.38	0.0–5.07	0.0	0.46
Culture/molecular	26	4124	21.52	18.60–24.44	97.9	0.000

^a^Different method of detection

^b^Number of distinctive prevalence values is reported

^c^Number of vegetable and fruit samples used to determine each estimate

#### Strength and weaknesses of this study

In few studies, the heterogeneity as high as 75%. This finding indicated a high proportion of heterogeneity to assess weighted mean between studies. Factors influencing variations that were not clarified in our study may have associated with this heterogeneity. This phenomenon is common for this kind of study due to limited number of published data. One of the limitations was due to English inclusion criteria, therefore other non-English reports were not included in our study. Data for most Oceania, Africa and South American countries were inadequate for analysis. As such, we were not able to estimate the prevalence of campylobacter in fresh vegetables among those countries.

The current systematic review and meta-analysis was the first study estimating the prevalence of *Campylobacter* in different kinds of fresh vegetables and fruits in various geographical areas. In addition the specific role of each species of bacteria was studied. The more applicable method of detection was also investigated.

## Conclusion

As final conclusion it seems that in spite of general low prevalence of the *Campylobacter* contamination in vegetable and fruits and the high level of consumption of these products raises it total risk of infection. Food chain is increasing the risk of contamination by different routes, for instances, primary production (the most effective one), postharvest contamination during transportation, food processing steps, packaging, distribution and cross contamination in the retail market are among the health hazards. Therefore, employing proper sanitation techniques is highly recommended during all the steps of food preparation.
